# PLEK2: a potential biomarker for metastasis and prognostic evaluation in uveal melanoma

**DOI:** 10.3389/fmed.2024.1507576

**Published:** 2024-12-02

**Authors:** Yichong Liu, Haiyue Wang, Qian Zhang, Xiaodi Gao, Yiqing Ji, Yuanzhang Zhu, Jingjing Zhang, Wenjuan Luo

**Affiliations:** ^1^Department of Ophthalmology, The Affiliated Hospital of Qingdao University, Qingdao, China; ^2^Department of Clinical Medicine, First College of Clinical Medicine, Binzhou Medical University, Yantai, China

**Keywords:** uveal melanoma, PLEK2, bioinformatics analysis, biomarker, differentially expressed genes

## Abstract

**Background:**

Uveal melanoma (UVM) is an aggressive tumor known for its high metastatic rate, making it necessary to delineate potential molecules that may promote the development of UVM. PLEK2 has been found to promote the progression and metastasis of some tumors, but its role in UVM has not yet been reported. Through this study, we hope to explore the effect of PLEK2 on the prognosis of UVM patients and to discover the potential functional role and intrinsic mechanism of PLEK2.

**Methods:**

The GEO datasets GSE211763 and GSE149920 were analyzed using GEO2R to identify differentially expressed genes that may be associated with UVM progression and metastasis. A Protein-Protein Interaction Network (PPI) was constructed to identify key molecules. The correlation between PLEK2 expression and overall survival was evaluated via GEPIA2, and clinical characteristics of UVM patients were compared based on PLEK2 levels. PLEK2 expression in UVM cell lines was assessed using the CCLE database and confirmed by qPCR and western blot. A weighted correlation network analysis (WGCNA) was performed, followed by gene ontology (GO) and Kyoto Encyclopedia of Genes and Genomes (KEGG) enrichment analyses. Finally, a search for miRNAs potentially regulating PLEK2 expression was performed using TargetScan, miRWalk, and TarBase databases.

**Results:**

Comparative analysis of the GEO datasets unveiled 79 commonly up-regulated genes and 238 commonly down-regulated genes. The PPI network identified 9 hub genes, with PLEK2 significantly linked to reduced overall survival. Clinical comparisons indicated significant differences in cancer status (*p* = 0.013) and tumor diameter (*p* = 0.039) between high and low PLEK2 expression groups. Elevated PLEK2 mRNA levels were confirmed in UVM cell lines compared to retinal pigment epithelial cells. PLEK2 was enriched in the calcium signaling pathway and associated with the Wnt/Ca2+ signaling pathway. A total of 21 miRNAs potentially regulating PLEK2 were predicted.

**Conclusion:**

PLEK2 is upregulated in UVM and correlates with poor patient prognosis, likely influencing the calcium signaling pathway. PLEK2 represents a promising prognostic biomarker and therapeutic target for UVM.

## 1 Introduction

Uveal melanoma (UVM) is the most prevalent primary intraocular malignancy in adults, varying greatly in frequency by age, race, and latitude, ranging from 0.1 to 8.6 per million (1). UVM originates from melanocytes in the iris, ciliary body, or choroid, and over 90% of uveal melanomas involve the choroid (2). Visual symptoms of UVM encompass blurred or distorted vision, visual field loss or photopsia, with patients typically being referred to an ocular oncologist by an optometrist, family doctor or ophthalmologist. The diagnosis is made primarily based on imaging modalities, including fundus photography, fluorescein angiography, fundus autofluorescence imaging, optical coherence tomography, and ultrasound imaging (3). Current therapeutic measures for UVM include radiation therapy, phototherapy, and surgery (4). Despite advances in the diagnosis and treatment of UVM over the past few decades, approximately 5% of patients are still accompanied by metastatic lesions at the time of first diagnosis (5), and ultimately approximately 50% of patients still develop metastases, most often involving the liver, metastasis-related deaths often occur within 1 year (4). Therefore, the suppression of tumor metastasis to prolong patient survival is of particular importance. Recent investigations indicate that activating mutations in the Gα11/Q pathway facilitate uveal melanoma oncogenesis, while alterations in the BAP1, SF3B1, or EIF1AX genes predict the progression of UVM toward metastasis (6). Considering the extremely poor prognostic outcome of UVM patients, the mechanisms of UVM occurrence and metastasis still require further study, necessitating the development of new biomarkers as prognostic indicators to enhance prognosis and individualized treatment.

Pleckstrin-2 (PLEK2) is located on chromosome 14q23.3-q24.1 and encodes a 353-amino acid protein. PLEK2 is a member of the Pleckstrin family and is widely expressed in multiple tissues, particularly in the thymus, stomach, large and small intestines, and prostate. It contains two PH domains at the N- and C-terminus, as well as a Disheveled, Egl-10, and Pleckstrin (DEP) domain in the center (7). PLEK2 has been shown to play a critical role in regulating many important biological processes, such as inflammation, erythropoiesis, tumorigenesis, and metastasis (8). Interestingly, PLEK2 binds to membrane-associated phosphatidylinositol produced by phosphatidylinositol 3-kinase, which subsequently redistributes the actin within cells and causes more microvilli and large lamellipodia with ruffle formation, inducing the cell spreading. This function may be closely related to the invasion and metastasis of tumors, and an increasing number of studies are now beginning to focus on the aspect of PLEK2 promoting tumor metastasis.

PLEK2 has been reported to be highly expressed in several tumor types and promotes cancer progression and metastasis, including breast cancer (9), gastric cancer (10), prostate cancer (11), gallbladder cancer (12), and non-small-cell lung cancer (13). Meanwhile, a comprehensive transcriptome analysis of whole blood identified PLEK2 as the most effective gene for differentiating CD45-subtype melanoma patients from the healthy population. Consequently, PLEK2 can be utilized for the early detection of melanoma (14). From a mechanistic standpoint, PLEK2 could interact with c-Myc and Akt to maintain the malignant phenotypes of tumors (12, 15).

The dysregulation of PLEK2 in tumors and its relationship with patients’ clinicopathological features and prognosis have been partially reported. Nevertheless, no study has yet explored the role of PLEK2 in UVM. RNA and DNA studies are an important part of biological and biomedical research and have been revolutionized with the development of microarray technology. In this study, we employed bioinformatics methods to identify PLEK2, a potential biomarker associated with tumorigenesis and metastasis in UVM and investigated the expression, prognostic value, and potential mechanisms of PLEK2 in UVM.

## 2 Materials and methods

### 2.1 Data download and organization

Microarray data for GSE211763 (16) and GSE149920 (17, 18) were downloaded from the National Center for Biotechnology Information (NCBI) and the Gene Expression Omnibus database (GEO^1^) (19). GSE211763 includes 5 groups of uveal melanoma tissues and 4 groups of adjacent tissues. GSE149920 includes 6 groups of metastatic uveal melanoma cell lines and 9 groups of primary uveal melanoma cell lines. In R software (version 4.3.2), the clinical data of 80 patients with UVM tumors in the TCGA database and their corresponding sequencing data were downloaded using the “TCGAbiolinks” package. The “dplyr” and “compareGroups” packages were used to organize the data. RNA-seq data of UVM tumor cell lines was obtained from the CCLE database.^2^

### 2.2 Differentially expressed genes (DEGs) screening

GEO2R^3^ is an interactive online tool designed to compare two or more datasets from the GEO series to identify DEGs under experimental conditions. Using a threshold of | logFC| > 1, a *P*-value < 0.05 was considered statistically significant, and DEGs were analyzed by GEO2R for 4 groups of uveal melanoma tissues and 4 groups of paraneoplastic tissues in GSE211763, as well as for 6 groups of metastatic uveal melanoma cell lines and 9 groups of primary uveal melanoma cell lines in GSE149920. Using the threshold of | logFC| > 1 and *P*-value < 0.05 as statistically significant, DEGs was analyzed by GEO2R for 4 groups of uveal melanoma tissues and 4 groups of paracarcinoma tissues in GSE211763, as well as for 6 groups of metastatic uveal melanoma cell lines and 9 groups of primary uveal melanoma cell lines in GSE149920. The two sets of differential genes were displayed via Venn diagrams using the “dplyr” and “VennDiagram” packages in the R software (version 4.3.2).

### 2.3 Construction protein–protein interaction (PPI) network and identification of core genes

The DEG PPI network was constructed and analyzed using the STRING online database^4^ with a composite score > 0.15, and the results were imported into Cytoscape to identify hub genes. The Cytohubba plugin in Cytoscape provides 11 topology analysis methods to sort nodes in a PPI network based on network characteristics and filter out hub genes.

### 2.4 Weighted correlation network analysis (WGCNA)

The RNA-seq data of 80 patients with UVM tumors were downloaded from TCGA, and the “WGCNA” and “tidyverse” packages were used to construct the gene co-expression network. The molecular expression data were logarithmically transformed into normally distributed data, outliers were excluded, and the recommended soft threshold was set to 3. A step-by-step method was used to construct the co-expression network, and the correlation between the gene modules and the clinical features was calculated.

### 2.5 Survival analysis and clinical correlation analysis

Correlation analysis between molecules and overall patient survival was conducted using the survival analysis module of GEPIA2,^5^ and the TIMER database^6^ was used to analyze PLEK2 expression in different types of tumors. Clinical data of 80 patients with UVM tumors were downloaded from TCGA, and clinical characteristics were extracted, including age, gender, person neoplasm cancer status, new tumor events, clinical stage, tumor basal diameter, and tumor thickness. 80 patients with UVM were divided into two groups based on PLEK2 expression, and the clinical characteristics of the two groups were compared.

### 2.6 Functional enrichment analysis

Gene ontology (GO) analysis was used to reveal the function of gene products from three aspects: biological process (BP), cellular component (CC), and molecular function (MF). KEGG Pathway enrichment analysis was performed using WebGestalt,^7^ and the top eight significantly enriched results were selected for visual presentation. Correlation analysis of PLEK2 with molecules of the Wnt/Ca2+ signaling pathway was conducted using the Correlation Analysis Module of GEPIA2, and a forest plot was drawn using the “ggplot2” package of the R software (version 4.3.2).

### 2.7 miRNA prediction

Online databases targetscan,^8^ miRWalk,^9^ and TarBase^10^ were used to predict potential miRNAs of PLEK2.

### 2.8 Cell culture

Human retinal pigment epithelial cells ARPE-19 and human ocular UVM cell line OCM1 were obtained from Beijing BeNa Culture Collection, C918 from Wuhan Procell Life Science & Technology Co., Ltd, OMM2.3, and MEL270 from Guangzhou Cellcook Biotech Co., Ltd. The ARPE-19 cell line was incubated in DMEM medium. C918, OCM-1, OMM2.3, and MEL270 cell lines were cultured in RPMI-1640. All the culture medium was added 10% fetal bovine serum (FBS) and 1% penicillin-streptomycin. After 48 h of continuous culture, all cell culture media were replaced with fresh media. When the cell fusion reached approximately 80%, the cells were digested and passaged for culture. Incubator settings: temperature (37°C), CO_2_ concentration in the incubator (5%), humidity (95%). The incubator was cleaned regularly and autoclaved mono-distilled water was changed.

### 2.9 Quantitative real time polymerase chain reaction (qPCR)

According to instructions, RNA was extracted utilizing RNA-easy Isolation Reagent (Vazyme, Nanjing, China). Subsequently, RNA was reserve transcribed by HiScript III RT SuperMix for qPCR (+gDNA wiper) (Vazyme, Nanjing, China) and amplified by qPCR. Relative mRNA expression of PLEK2 was estimated by the method of comparative amplification cycles using GAPDH as the internal reference. The 2^–ΔΔCT^ method was used for data analysis, and three independent qPCR experiments were performed. Utilized primers are as follows: Human-PLEK2-F: 5′-GCGCGATGGTTCATCCTT-3′, Human-PLEK2-R: 5′-AATGAGGAGCGGTCGGTTTT-3′; Human-GAPDH-F: 5′-AGGTCGGTGTGAACGGATTTG-3′, Human-GAPDH-R: 5′-TGTAGACCATGTAGTTGAGGTCA-3′.

### 2.10 Western blot

Whole-cell lysates were extracted using RIPA lysis buffer (Beyotime) on ice for 30 min. Then the supernatants were collected by centrifugation. The total protein concentration was measured with a BCA kit according to the instructions of manufacturer. The protein samples were then subjected to separation via SDS-PAGE before being transferred onto PVDF membranes. These membranes were subsequently blocked with 5% non-fat milk at room temperature for a period of two hours. Primary antibodies were incubated overnight at 4°C, while the secondary antibodies were incubated for 1 h at room temperature. The primary antibodies employed in this study included anti-PLEK2 (Proteintech), anti-β-tubulin (Proteintech), and the secondary antibody (Elabscience). The immunoreactive signals were detected using the ECL Luminescence Reagent (Meilunbio, Dalian, China), ensuring a clear and precise visualization of the protein bands.

### 2.11 Statistics analysis

All experiments were repeated at least three times unless otherwise noted. Experimental data were statistically analyzed using a two-sided unpaired *t*-test in Prism 9.0 (GraphPad, USA), and the data were presented as the mean ± SEM (standard error of the mean). Chi-square test/Fisher’s exact probability method was used to analyze the relationship between PLEK2 expression and clinical features. Spearman Correlation Test was used for correlation analysis between PLEK2 and Wnt/Ca2+ pathway molecules. *P < 0.05 was considered to have statistical significance.

## 3 Results

### 3.1 Identification of genes associated with the development and metastasis of UVM

**FIGURE 1:**
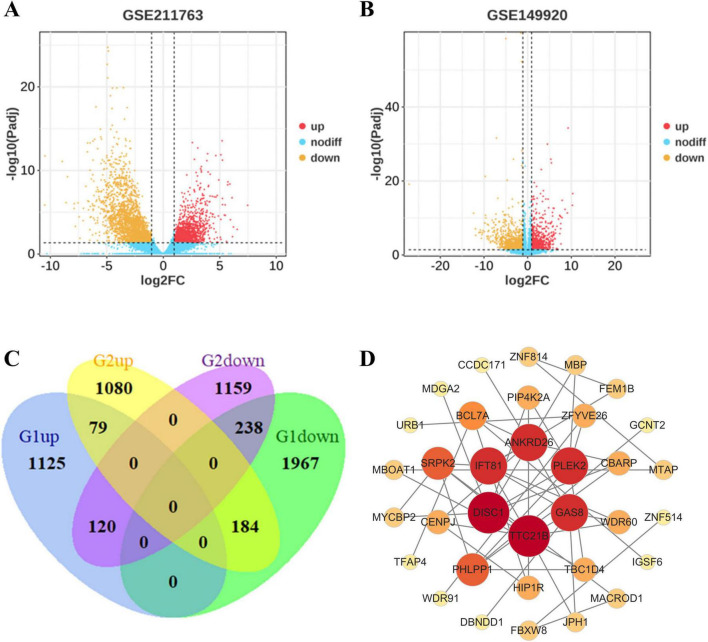
Identification of genes associated with development and metastasis of UVM. **(A,B)** Volcano plot of DEGs in each dataset. Red dots: significantly upregulated genes in UVM; Yellow dots: significantly downregulated genes in UVM; Blue dots: non-differentially expressed genes. **(C)** Venn diagram of overlapping DEGs from GSE211763 and GSE149920 datasets. G1 represents the gene in the GSE211763 dataset; G2 represents the gene in the GSE149920 dataset. **(D)** PPI network of upregulated genes.

The differentially expressed genes between UVM tissues and paracancerous tissues in dataset GSE211763 and between metastatic and primary UVM in dataset GSE149920 were obtained by GEO2R. Threshold | logFC| > 1 and *P* < 0.05 was set for the two gene sets (Figures 1A, B) and the intersection was taken (Figure 1C), yielding 79 differentially expressed genes that were commonly upregulated. We hypothesized that these genes were associated with the occurrence and metastasis of UVM.

A PPI network was constructed using the STRING database to predict the association of these DEGs at the protein level, setting the composite score > 0.15 To further identify the hub genes, we applied Cytohubba plugin of Cytoscape to rank the top 10 nodes in the above PPI network according to four topological analysis methods, including MCC, MNC, Degree, and EPC (Table 1). A total of 9 overlapping hub genes were determined for further analysis, namely PLEK2, TTC21B, GAS8, IFT81, DISC1, WDR60, ANKRD26, SRPK2 and PHLPP1 (Figure 1D).

**TABLE 1:** Highly differentiated expressed genes ranked in Cytohubba plugin of Cytoscape.

	Rank methods in cytoHubba			
**Catelogy**	**MCC**	**MNC**	**Degree**	**EPC**
Gene symbol top 10	***TTC21B***	***TTC21B***	***TTC21B***	***TTC21B***
	***GAS8***	***GAS8***	***DISC1***	***DISC1***
	***IFT81***	***IFT81***	***GAS8***	***IFT81***
	***DISC1***	***DISC1***	***IFT81***	***GAS8***
	***PLEK2***	***PLEK2***	***PLEK2***	***ANKRD26***
	***WDR60***	***WDR60***	***ANKRD26***	***PLEK2***
	***ANKRD26***	***ANKRD26***	***SRPK2***	***SRPK2***
	***SRPK2***	***SRPK2***	***PHLPP1***	***PHLPP1***
	***PHLPP1***	***PHLPP1***	*BCL7A*	*CBARP*
	*BCL7A*	*TBC1D4*	***WDR60***	***WDR60***

The bold text indicates the hub genes common to all four topological analysis methods.

### 3.2 PLEK2 was correlated with the overall survival time of UVM patients

**FIGURE 2:**
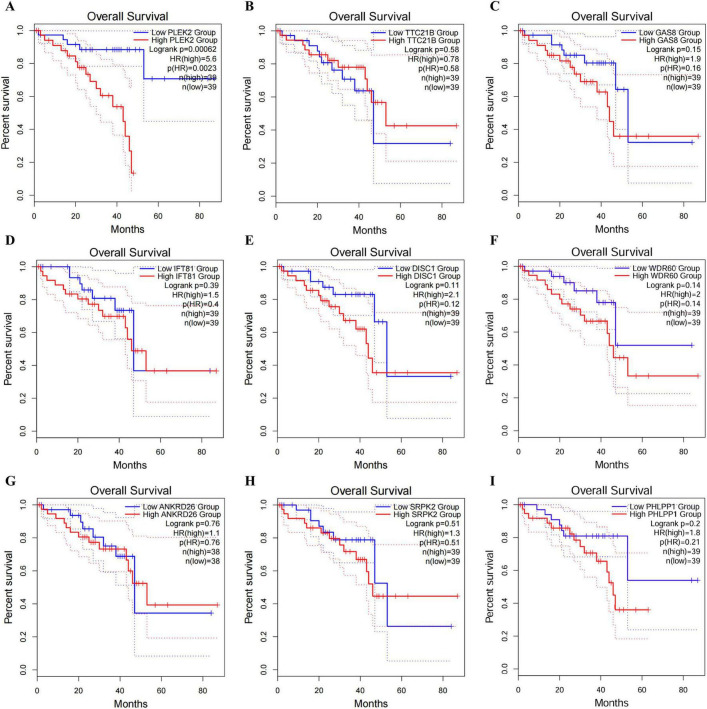
Survival analysis of nine Hub genes in UVM. **(A–I)** Kaplan-Meier survival curve of PLEK2, TTC21B, GAS8, IFT81, DISC1, WDR60, ANKRD26, SRPK2, and PHLPP1 in UVM patients.

Survival analysis of nine hub genes in UVM patients using GEPIA2 database revealed that higher PLEK2 expression was significantly associated with shorter overall survival time of UVM patients; thus, PLEK2 was selected for further investigation (Figures 2A–I).

### 3.3 PLEK2 was upregulated in UVM cell lines

**FIGURE 3:**
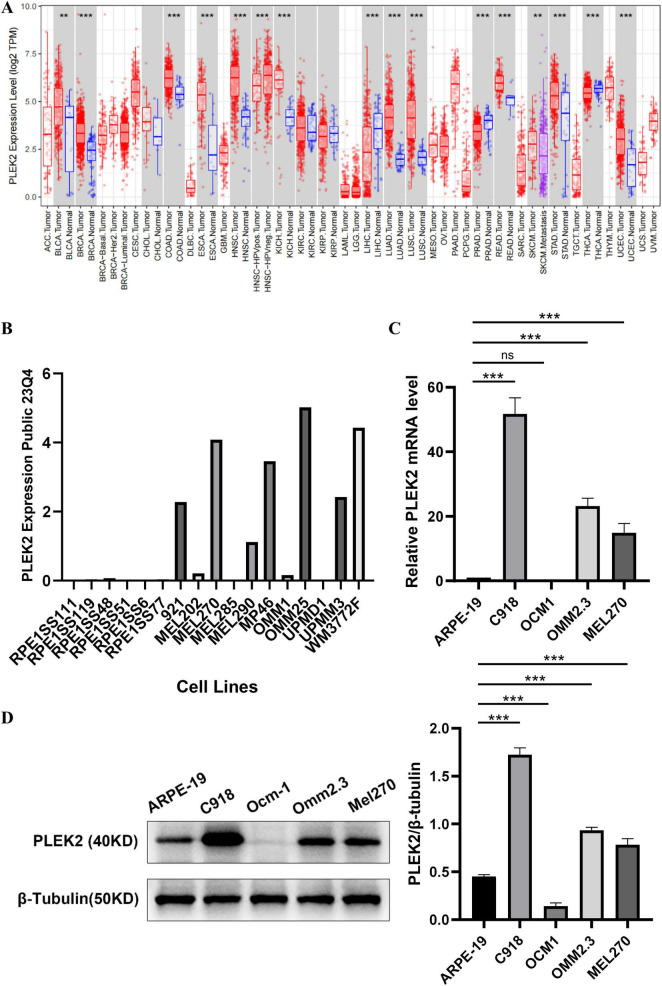
PLEK2 expression was increased in UVM cell lines. **(A)** PLEK2 mRNA level in various tumor and control tissues. **(B)** PLEK2 mRNA levels in multiple cell lines. RPE1SS111, RPE1SS119, RPE1SS48, RPE1SS51, RPE1SS6, and RPE1SS77 were retinal pigment epithelial cell lines. 921, MEL202, MEL270, MEL285, MEL290, MP46, OMM1, OMM25, UPMD1, UPMM3 and WM3772F were UVM tumor cell lines. **(C)** PLEK2 mRNA level was detected by qPCR in ARPE and tumor cell lines, and GAPDH was used as an internal control. **(D)** PLEK2 protein level was detected by western blot in ARPE and tumor cell lines. Data from at least three independent samples were represented as mean ± SEM; *P < 0.05, **P < 0.01, ***P < 0.001.

The expression of PLEK2 in different tumor types was analyzed using the online tool TIMER, which demonstrated that PLEK2 expression was generally elevated in a wide range of tumors (Figure 3A). Subsequently, PLEK2 expression in UVM cell lines was analyzed using the CCLE database, which demonstrated that UVM cell lines exhibited higher PLEK2 expression levels compared to retinal pigment epithelial cells (Figure 3B). Furthermore, qPCR and western blot results also indicated significantly higher expression of PLEK2 in UVM cell lines, including C918, OMM2.3, and MEL270, compared to the ARPE cell line (Figures 3C, D).

### 3.4 PLEK2 was correlated with poorer clinical characteristics in UVM patients

In order to investigate the relationship between PLEK2 and other clinical characteristics of UVM patients, 80 UVM patients’ clinical data were downloaded from TCGA and divided into high-expression (40 cases) and low-expression (40 cases) groups based on PLEK2 level. There was a statistically significant difference between the two groups in person neoplasm cancer status (*p* = 0.013) as well as tumor basal diameter (*p* = 0.039), indicating higher PLEK2 expression was associated with worse clinical characteristics (Table 2).

**TABLE 2:** Correlation of PLEK2 expression and clinical characteristics of UVM patients.

	[All]	High PLEK2 level	Low PLEK2 level	p-value overall
	***N* = 80**	***N* = 40**	***N* = 40**	
Age	61.6 (13.9)	60.0 (12.8)	63.4 (15.0)	0.278
Gender				0.652
Female	35 (43.8%)	16 (40.0%)	19 (47.5%)	
Male	45 (56.2%)	24 (60.0%)	21 (52.5%)	
person_neoplasm_cancer_status:				0.013
Tumor free	61 (77.2%)	25 (64.1%)	36 (90.0%)	
With tumor	18 (22.8%)	14 (35.9%)	4 (10.0%)	
new_tumor_events:				0.163
No	51 (63.7%)	22 (55.0%)	29 (72.5%)	
Yes	29 (36.2%)	18 (45.0%)	11 (27.5%)	
clinicial_stage:				0.090
Stage II	36 (45.0%)	15 (37.5%)	21 (52.5%)	
Stage III	40 (50.0%)	21 (52.5%)	19 (47.5%)	
Stage IV	4 (5.00%)	4 (10.0%)	0 (0.00%)	
tumor_basal_diameter	16.9 (3.45)	17.7 (3.50)	16.1 (3.25)	0.039
tumor_thickness	10.4 (2.81)	10.3 (3.09)	10.5 (2.54)	0.708

### 3.5 PLEK2 may be associated with the Wnt/Ca2+ signaling pathway

**FIGURE 4:**
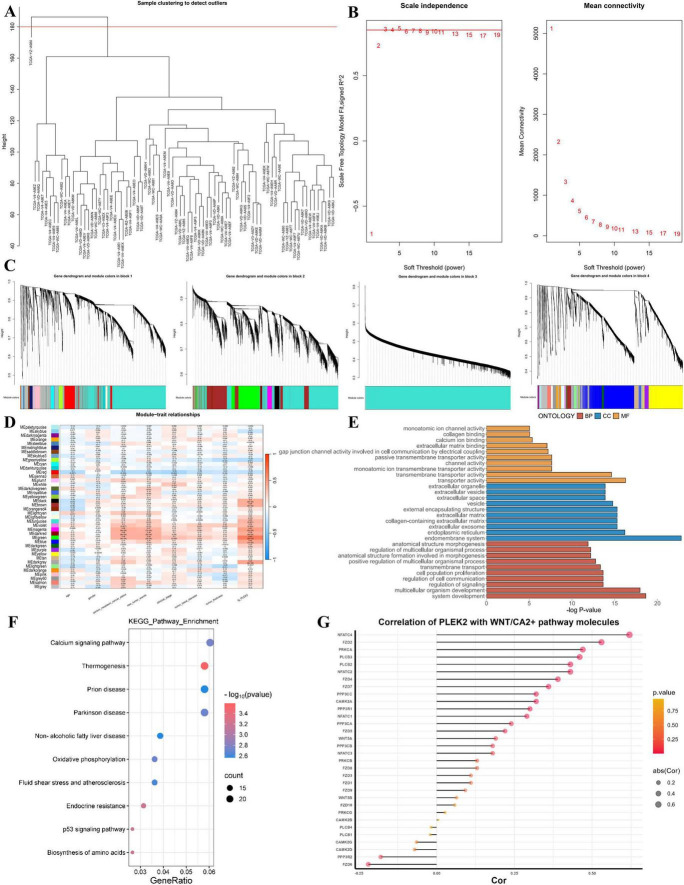
Bioinformatics analysis seeks the potential function of PLEK2. **(A–C)** Construction of gene co-expression network, Sample selection **(A)**, Determine the appropriate soft threshold **(B)** and Gene clustering tree **(C)**, molecules with similar expression pattern were grouped into a module, with each color representing one module. **(D)** Heat map of correlation between different color modules and patients’ clinical features as well as PLEK2 expression. **(E)** GO enrichment results of green module genes. **(F)** Enrichment results of KEGG pathway of green module genes. **(G)** The correlation between PLEK2 and Wnt/Ca2+ pathway factors.

In order to explore the potential function of PLEK2 in UVM, we downloaded the sequencing data of 80 UVM patients from TCGA database, constructed the WGCNA gene co-expression network, searched for gene molecules that had similar expression patterns with PLEK2, and performed KEGG pathway enrichment analysis by WebGestalt (Figure 4A). After removing one outlier sample, the optimal soft threshold was 3 (Figure 4B). Molecules were divided into different modules using a stepwise approach, correlation between these gene modules and clinical characteristics of UVM patients was analyzed (Figure 4C), the results showed that MEgreen, MEmagenta, MEdarkred, and MEviolet modules were significantly correlated with new tumor events as well as PLEK2 expression (Figure 4D). The gene module MEgreen containing PLEK2 was analyzed for GO enrichment and the first ten entries were shown. In Biological Process, PLEK2 was significantly associated with transmembrane transporter activity and transporter activity. In terms of Cellular Components, PLEK2 was particularly enriched in endoplasmic reticulum and endomembrane system. In terms of Molecular Function, PLEK2 was significantly associated with multicellular organism development and system development (Figure 4E). KEGG pathway enrichment was performed by WebGestalt for the gene module MEgreen, and the first ten entries were shown, among which Calcium signaling pathway was significantly enriched (Figure 4F). Considering that the tumor-promoting effects of the Wnt/Ca2+ signaling pathway have received increasingly widespread attention, we further analyzed the correlation between PLEK2 and molecules in Wnt/Ca2+ signaling pathway using GEPIA2. The results indicate a general correlation between PLEK2 expression and Wnt/Ca2+ signaling pathway molecules in the context of UVM (Figure 4G).

### 3.6 PLEK2 upstream miRNA prediction

**FIGURE 5:**
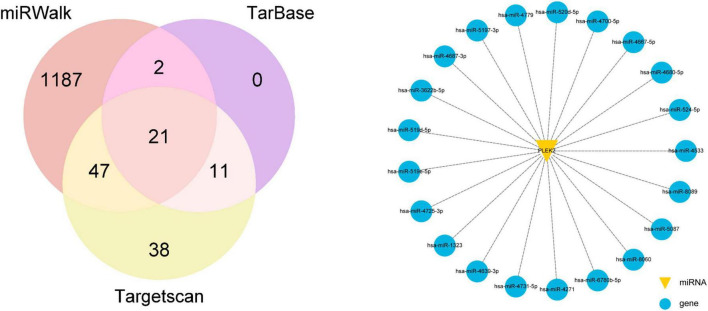
Predicted miRNAs targeting PLEK2.

Finally, we determined to explore the upstream regulatory mechanisms of PLEK2. miRNAs usually regulate target gene mRNA levels by recognizing and binding to mRNA sequences with the help of RISC (RNA induced silencing complex) silencing complex. The miRNAs that may regulate PLEK2 were predicted by TargetScan, miRWalk, and TarBase online databases, and 21 miRNAs were predicted jointly by the three databases (Figure 5).

## 4 Discussion

UVM is a highly malignant cancer, and despite some advancement, the current understanding of UVM pathophysiology has not significantly enhanced patient survival rates. Hematogenous metastasis through the intraocular venous system can occur early in UVM, and up to 50% of patients eventually develop metastatic disease, which would cause death usually within a year (1). Thus, identifying biomarkers predictive of UVM metastasis is crucial.

In this study, microarray datasets of GSE211760 and GSE149920 were utilized to identify DEGs implicated in UVM progression and metastasis, revealing 79 commonly upregulated genes. Subsequently, the PPI network was further analyzed, resulting in the identification of 9 hub genes. It was found that high expression of PLEK2 was correlated with a shorter overall survival. Consequently, PLEK2 was thus selected for further investigation.

Due to the rarity of UVM, pathological tissues are scarce and existing public databases lack information on paracancerous samples. We analyzed PLEK2 expression in tumor tissues using the TIMER database, which showed elevated PLEK2 levels across various tumor types compared to control tissues. To examine PLEK2 expression in UVM at the cellular level, we compared UVM cell lines and retinal pigment epithelial cell lines from the CCLE database. The results indicated that the expression of PLEK2 was significantly higher in the UVM cell lines than in the retinal pigment epithelial cell lines. qPCR and western blot results demonstrated that PLEK2 expression in UVM cell lines C918, OMM2.3, and MEL270 was significantly higher than in ARPE19, consistent with our expectations. Mutations in GNAQ/GNA11 are closely associated with the tumorigenesis of UVM. Interestingly, both the OMM2.3 and MEL270 cell lines exhibit GNAQ mutations (626 A > C), whereas the OCM1 cell line, which has low PLEK2 expression, shows no such mutations (20). Furthermore, PLEK2 expression is highest in the highly invasive C918 cell line. These findings further suggest that PLEK2 may be linked to the development and metastasis of UVM.

To evaluate the association between PLEK2 and clinical characteristics of UVM patients, we compared clinical data from patients with high and low PLEK2 expression. Significant differences were noted in cancer status and tumor basal diameter, indicating that high PLEK2 expression is linked to poorer clinical outcomes in UVM patients.

Primary benign solid tumors may remain dormant for extended periods. However, when they are continually exposed to the selective pressures of immunity and the microenvironment, the tumor cells divide at a much faster frequency to survive. Ultimately the key event that accelerates the mortality rate of the patient is metastasis (21). For distant metastasis, primary tumor cells must invade the mesenchymal environment, infiltrate, and spread through the vasculature, or seed distant organs (22). Epithelial-mesenchymal transition (EMT) is a reversible process in which epithelial cells lose their own identity and become mesenchymal cells, which is an important part of normal embryonic development and tissue regeneration. However, in cancer progression and metastasis, aberrant activation of EMT promotes tumor metastasis, increases tumor stemness, and enhances resistance to chemotherapy and immunotherapy (23). It has recently been demonstrated that various factors, including ZNF704 (24), PRRX1 (25), and CD146 (26), can promote UVM invasion and migration through activation of EMT, thereby suggesting that EMT plays an important role in UVM metastasis. In addition, disruption of the vascular endothelial barrier is an important condition for cancer cell invasion into the vasculature, and endothelial-mesenchymal transition (EndoMT) of vascular endothelial cells is an important mechanism of barrier disruption (13). Endothelial cells (ECs) share numerous characteristics with epithelial cells, including strong apical-basal polarity, the capacity to form tubes, and the potential to transform into mesenchymal-like cells. Consequently, EndoMT is closely related to EMT and shares a number of key transcription factors, including Snail, Slug, Twist, Zeb1, and Zeb2. A study reported that PLEK2 was highly expressed in non-small cell lung cancer cells and promoted EMT, EndoMT, vascular endothelial barrier disruption, and tumor metastasis, suggesting the role of PLEK2 in tumor metastasis (13). The current research on PLEK2 has focused on head and neck squamous cell carcinoma, esophageal squamous cell carcinoma, gallbladder cancer, myeloproliferative neoplasms, gastric cancer, non-small cell lung cancer, and breast cancer. However, the involvement of PLEK2 in UVM remains unexplored. By investigating the potential role and underlying mechanism of PLEK2 in UVM, our objective is to identify more efficacious therapeutic interventions for this malignancy.

To delve deeper into the potential function of PLEK2 in UVM, we constructed a WGCNA gene co-expression network to identify genes exhibiting a similar expression pattern with PLEK2. Subsequent GO enrichment analysis was conducted to elucidate the biological functions and signaling pathways of these co-expressed genes. KEGG pathway enrichment analysis facilitated through the WebGestalt platform revealed significant enrichment in the Calcium signaling pathway. The canonical Wnt/β-catenin signaling pathway has been extensively studied in oncogenesis, with accumulating evidence indicating that dysregulation of this pathway contributes to the development and progression of various solid tumors and hematologic malignancies (27–33). Interest has been growing in the Wnt/Ca2+ pathway as an atypical Wnt pathway in tumorigenesis. Previously, our research reported that knockdown of Wnt5a with siRNA to inhibit the Wnt5a/CaMKII pathway resulted in blockage of angiogenic mimicry formation and inhibition of expression of angiogenesis-related factors such as HIF-1a, VEGFR2, PDGFR, VEGFA, VE-calmodulin, and EphA2 (34), suggesting a pro-angiogenic role of Wnt/CaMKII in UVM. In addition, many studies have shown that the Wnt/Ca2+ pathway may be associated with tumor metastasis and EMT. WIF1 reduced glioblastoma migration *in vivo* and *in vitro* by inhibiting the Wnt5a/p38-MAPK/Ca2+ pathway (35). ATF-2 activated the Wnt/Ca2+ pathway and promoted the expression of Wnt5a, Wnt11, CaMKII and NLK, which regulated the proliferation and invasion of non-small cell lung cancer cells (36), suggesting that Ca signaling is directly involved in malignant behavior of tumor cells. Interestingly, we found a general correlation between PLEK2 expression and Wnt/Ca2+ signaling pathway molecules in UVM by GEPIA2 correlation analysis, in which NFATC4 had the highest correlation. Abnormally activated NFATC4 has been reported to be involved in and regulate the initiation, proliferation, invasion, and metastasis of a variety of cancers, including lung, breast, ovarian, cervical, skin, liver, and pancreatic cancers, as well as glioma, primary myelofibrosis, and acute myeloid leukemia (37), and it is worth further investigating whether PLEK2 exerts a pro-carcinogenic effect by regulating NFATC4.

miRNAs are small non-coding RNAs (ncRNAs), approximately 22 nucleotides in length, that play a crucial role in mRNA regulation, and their dysregulation has been implicated in a wide range of human diseases, including cancer. Typically, miRNAs bind to the mRNA 3′-untranslated region (3′-UTR) of target mRNAs through base-pair complementarity, inhibiting translation or directing mRNA degradation, thereby suppressing protein synthesis (38). miRNAs have also been implicated in UVM progression. It has been reported that miR-20a is expressed at significantly higher levels in UVM cells and tissues and promotes the proliferation, invasion, and migration ability of UVM cells (39). miR-92a-3p can decrease the susceptibility of UM cells to TNF-related apoptosis-inducing ligand (TRAIL)-mediated apoptosis by directly downregulating MYC-binding protein 2 (MYCBP2) (40). Additionally, certain miRNAs can also exert inhibitory effects on the progression of UVM. miR-204 acts to inhibit the migration and invasive capabilities of UVM cells by downregulating the expression of RAB22A (41). miRNA-130a inactivates the Wnt/β-catenin signaling pathway by downregulating USP6, thereby suppressing the metastasis of UVM cells (42). All these results suggest that miRNAs are involved in UVM progression, including cell cycle, cell proliferation, apoptosis, and tumor invasion, and play important and complex roles in the regulatory network of UVM. We predicted several miRNAs based on databases, in anticipation of finding upstream miRNA molecules that may regulate PLEK2 expression.

In conclusion, our extensive bioinformatics analysis has revealed that PLEK2 expression is elevated in UVM, suggesting its potential as a novel diagnostic and prognostic biomarker for UVM. However, there are notable limitations to our study. Primarily, the lack of experimental validation stands out as the most significant limitation. Additionally, the mechanism by which PLEK2 influences tumorigenesis and progression in UVM remains to be elucidated. Consequently, further investigations are necessary to clarify the functions and underlying mechanisms of PLEK2.

## Data Availability

The original contributions presented in this study are publicly available. This data can be found here: https://figshare.com/s/5b2baa7457f3adb5ba37.

## Footnotes

1
http://www.ncbi.nlm.nih.gov/geo/

2
https://sites.broadinstitute.org/ccle

3
http://www.ncbi.nlm.nih.gov/geo/geo2r

4
https://string-db.org/

5
http://gepia2.cancer-pku.cn/

6
https://cistrome.shinyapps.io/timer/

7
https://www.webgestalt.org/

8
www.targetscan.org

9
http://mirwalk.umm.uni-heidelberg.de

10
https://dianalab.e-ce.uth.gr/tarbasev9
