# Supplementary material for: PLEK2: a potential biomarker for metastasis and prognostic evaluation in uveal melanoma
Source: Front Med (Lausanne). 2024 Dec 2;11:1507576. doi: 10.3389/fmed.2024.1507576 (PMC11646761; doi:10.3389/fmed.2024.1507576)
Supplement: Supplementary file 1 [file Data_Sheet_1.zip › Supplementary Material/Figure2/Figure2.docx]

Figure2 was performed using the survival analysis module of GEPIA2 (http://gepia2.cancer-pku.cn/).
