# Supplementary material for: PLEK2: a potential biomarker for metastasis and prognostic evaluation in uveal melanoma
Source: Front Med (Lausanne). 2024 Dec 2;11:1507576. doi: 10.3389/fmed.2024.1507576 (PMC11646761; doi:10.3389/fmed.2024.1507576)
Supplement: Supplementary file 1 [file Data_Sheet_1.zip › Supplementary Material/Figure3/Figure3A,B.docx]

Figure3A: The expression of PLEK2 in different tumor types was analyzed using the online tool TIMER (<https://cistrome.shinyapps.io/timer/>).

Figure3B: RNA-seq data of UVM tumor cell lines were obtained from the CCLE database (<https://sites.broadinstitute.org/ccle>).
