# Supplementary material for: PLEK2: a potential biomarker for metastasis and prognostic evaluation in uveal melanoma
Source: Front Med (Lausanne). 2024 Dec 2;11:1507576. doi: 10.3389/fmed.2024.1507576 (PMC11646761; doi:10.3389/fmed.2024.1507576)
Supplement: Supplementary file 1 [file Data_Sheet_1.zip › Supplementary Material/Figure4/Figure4.docx]

Figure4A-E were all generated by R.

Figure4F is plotted from the enrichment results generated by R.

Figure4G: We used GEPIA2 to analyze the correlation between PLEK2 and molecules in the WNT/Ca2+ signaling pathway before mapping the Figure.
